# Brain structure, IQ, and psychopathology in young offspring of patients with schizophrenia or bipolar disorder

**DOI:** 10.1192/j.eurpsy.2019.19

**Published:** 2020-01-31

**Authors:** Neeltje E.M. van Haren, Nikita Setiaman, Martijn G.J.C. Koevoets, Heleen Baalbergen, Rene S. Kahn, Manon H.J. Hillegers

**Affiliations:** 1 Department of Psychiatry, University Medical Center Utrecht Brain Center, Utrecht, The Netherlands; 2 Department of Child and Adolescent Psychiatry/Psychology, Erasmus University Medical Center, Sophia Children’s Hospital, Rotterdam, The Netherlands; 3 Department of Psychiatry, Icahn School of Medicine at Mount Sinai, New York, New York, USA

**Keywords:** Bipolar disorder, neurodevelopment, offspring, schizophrenia, structural magnetic resonance imaging

## Abstract

**Background.:**

Studying offspring of schizophrenia (SZo) and bipolar disorder patients (BDo) provides important information on the putative neurodevelopmental trajectories underlying development toward severe mental illnesses. We compared intracranial volume (ICV), as a marker for neurodevelopment, and global and local brain measures between SZo or BDo and control offspring (Co) in relation to IQ and psychopathology.

**Methods.:**

T1-weighted magnetic resonance imaging (MRI) brain scans were obtained from 146 participants (8–19 years; 40 SZo, 66 BDo, 40 Co). Linear mixed models were applied to compare ICV, global, and local brain measures between groups. To investigate the effect of ICV, IQ (four subtests Wechsler Intelligence Scale for Children/Wechsler Adult Intelligence Scale-III) or presence of psychopathology these variables were each added to the model.

**Results.:**

SZo and BDo had significantly lower IQ and more often met criteria for a lifetime psychiatric disorder than Co. ICV was significantly smaller in SZo than in BDo (*d* = −0.56) and Co (*d* = −0.59), which was largely independent of IQ (respectively, *d* = −0.54 and *d* = −0.35). After ICV correction, the cortex was significantly thinner in SZo than in BDo (*d* = −0.42) and Co (*d* = −0.75) and lateral ventricles were larger in BDo than in Co (*d* = 0.55). Correction for IQ or lifetime psychiatric diagnosis did not change these findings.

**Conclusions.:**

Despite sharing a lower IQ and a higher prevalence of psychiatric disorders, brain abnormalities in BDo appear less pronounced (but are not absent) than in SZo. Lower ICV in SZo implies that familial risk for schizophrenia has a stronger association with stunted early brain development than familial risk for bipolar disorder.

## Introduction

Offspring of parents with severe mental illnesses (SMI), such as schizophrenia (SZ) and bipolar disorder (BD), have a more than 10-fold increased risk to develop a psychotic disorder or BD and a twofold to threefold increased risk to develop any lifetime DSM-IV axis-I disorder compared to offspring of parents without these diagnoses [1]. Importantly, in contrast to adult relatives of patients with SMI, adolescent offspring are below the usual age of onset, that is, young adulthood. Moreover, offspring are often (still) unmedicated, even when (subtle) symptoms are present. Consequently, the intake of psychotropic medication does not influence the clinical, cognitive, and neural measures and does not confound differences between individuals with and without specific symptoms or diagnoses. It has also been suggested that SMI may be a result of (early) neurodevelopmental abnormalities [2,3]. Thus, adolescent offspring of patients with SZ (SZo) and BD (BDo) provide a unique population to investigate putative neurodevelopmental trajectories toward these disorders.

Intracranial volume (ICV) may be considered a neurodevelopmental marker in early life and possibly early adolescence. Around the age of 5, ICV has reached 90% of its full size, implying that the size of the cranium is mostly determined by the growth of the brain during these early years [4,5]. Another spurt occurs between 10 and 15 years of age [4]. Moreover, the adolescent brain undergoes significant structural and functional development, creating a sensitive window for psychiatric symptoms [6], which makes adolescence a particularly interesting time window to study.

Interestingly, a smaller ICV relative to controls is consistently found in adult patients with SZ, but not in patients with BD [7–10]. Investigating ICV in SZo and BDo may shed light on the disease-specific neurodevelopmental nature of SMI that emerge later in life.

In addition to ICV, global and local cortical brain measures show abnormalities in SZ and BD patients [7–10], with usually lower effect sizes in BD patients. A recent meta-analysis comparing first-degree relatives to controls (which included the current sample; [11]) reported larger ICV in BD relatives and no difference in SZ relatives. Interestingly, smaller global brain volumes were found after correction for ICV in SZ relatives, but not in BD relatives. However, De Zwarte et al. [11] did not correct for the possible confounding effect of IQ, which is considered an indication of aberrant neurodevelopment preceding the onset of SMI, in particular for SZ [12]. A lower premorbid IQ is found in SZ patients, while premorbid IQ appears to be higher in BD patients [13], although findings are inconsistent [14,15]. Moreover, the presence of (subclinical) psychopathology during adolescence is a well-established premorbid indicator of risk for SMI later in life. Both IQ and psychopathology have been found to be associated with ICV and global and local brain measures in SZ and BD [9,11,13,16–23].

The aim of the current study is threefold. First, we compare ICV, IQ, and presence of psychopathology among SZo, BDo, and control offspring (Co) to investigate whether we can identify evidence for abnormal neurodevelopment in SZo and/or BDo. As findings of smaller ICV are more consistently found in SZ than in BD patients [7–10] and findings that lower IQ increases risk for SZ are more consistently found than for BD [12], we hypothesize that ICV is smaller and IQ is lower in SZ, but not in BDo, as compared to Co. In line with previous studies [1], we expect that presence of psychopathology is increased in SZo and BDo compared to Co. Second, we compare global (i.e., total brain, gray matter [GM], white matter [WM], and cerebellar volumes, mean cortical thickness and mean surface area) and local (i.e., subcortical and cortical Region of Interest [ROI] volumes) brain measures between groups, with and without ICV correction. Based on previous offspring studies [24–34], we expect that brain abnormalities are more pronounced in SZo than in BDo relative to Co. Finally, we investigate whether IQ or presence of psychopathology explains potential differences between groups in global and local structural brain measures. Both may explain some variation in brain abnormality differences between the groups; however, in line with a recent meta-analysis [11], we hypothesize that smaller brain measures will be significantly related to familial risk.

## Methods

### Participants

A total of 146 children and adolescents participated between 2010 and 2015 in the Dutch Bipolar and Schizophrenia Offspring study at baseline, that is, 40 SZo (8.9–18.9 years), 66 BDo (9.1–19.8 years), and 40 Co (8.2–17.2 years). Brain imaging, genetic, cognitive, psychological, and environmental data were collected on several visits in the University Medical Center Utrecht (UMCU). Co were recruited via advertisement on schools or leisure clubs (57.5%), or via hospital staff (42.5%). SZo and BDo were recruited via their psychiatrist from the UMCU or other mental health care centers in the Netherlands (28.3%), their parent’s or sibling’s psychiatrist (37.7%), or through advertisement (34%; e.g., lectures, patient advocacy groups and study website). Inclusion criteria were offspring who were Dutch speaking and specifically for the at-risk offspring, at least one first- or two second-degree family members (with the vast majority being offspring). Exclusion criteria were severe physical illness, handicap, neurological problems (e.g., open- or closed head injury, epilepsy) or IQ < 70, and specifically for Co, a first-degree relative with a severe mood or psychotic disorder. Co were not excluded in case one of the parents has had a single mild depressive episode during young adulthood to prevent the inclusion of a super healthy population.

Recruitment of particularly SZo is difficult, thus groups were not matched on age, sex, and IQ. Groups were matched on parental level of education.

Written informed consent was obtained from offspring older than 12 years, and both parents or legal caregivers for the participation of offspring aged 8–18 years. Parents also gave written consent for their own participation. The Medical Ethics Committee of the UMCU approved the study.

### MRI acquisition

Magnetic Resonance Imaging (MRI) brain scans were obtained on a Philips 3-Tesla Achieva scanner (Philips Medical Systems, Best, the Netherlands), located at the UMCU. A three-dimensional T1-weighted sagittal spoiled-gradient fast-field echo scan of the whole brain (with 200 contiguous slices; 0.75 mm × 0.75 mm × 0.8 mm voxels, TE = 4.6 ms, TR = 10 ms, flip angle = 8°, field of view = 240 mm) was acquired. All images were coded to ensure blinding for participant identification and diagnoses during image processing.

### Image processing

The automated segmentation procedure of FreeSurfer software package version 5.3.0 (http://surfer.nmr.mgh.harvard.edu/) was used for subcortical and cortical volume, cortical thickness, and cortical surface area estimations. See the supplementary text for a detailed description of the processing procedure. After image processing, a quality check for volume, thickness, and surface labeling was performed according to the ENIGMA criteria (http://enigma.ini.usc.edu/). Manual corrections were made in case of poor GM, WM, or cerebral spinal fluid differentiation. Fifteen images (*N*_Co_ = 3, *N*_BDo_ = 11, and *N*_SZo_ = 1) with poor quality of ROI labeling were excluded from the analyses, resulting in 40 Co, 66 BDo, and 40 SZo.

### Instruments

All psychiatric and neuropsychological assessments were conducted by trained interviewers with a bachelor’s or master’s level degree in medicine or psychology, supervised by a psychiatrist certified in adult, and child and adolescent psychiatry (M.H.J.H.).

#### Parents

Index parents (i.e., the parent with SZ or BD) received a Structured Clinical Interview for DSM-IV Axis I Disorders (SCID-I; [35]) to confirm their DSM-IV [36] axis I diagnoses. Co-parents and control parents were screened for lifetime psychopathology with the mini-Schedule for Clinical Assessment in Neuropsychiatry (mini-SCAN) interview [37], followed by a SCID-I in case of reported psychopathology.

#### Offspring

Offspring received the Schedule for Affective Disorders and Schizophrenia for School Age Children Present and Lifetime Version (K-SADS-PL; [38]), which is a face-to-face semi-structured interview. The interview was administered to participants and their parents separately and summary information was used to establish lifetime DSM-IV axis I diagnoses. Autism spectrum diagnoses were based on psychiatric evaluations from the outpatient clinic or referring psychiatrist. In addition, autism spectrum symptoms were evaluated with an additional supplement to the K-SADS-PL according to the DSM-5.

IQ was estimated based on the subtests Picture Arrangement, Block Design, Vocabulary, and Information from the Wechsler Intelligence Scale for Children-III [39] for participants younger than 17 years old or Wechsler Adult Intelligence Scale-III [40] from the age of 17.

### Statistics

#### Demographic variables

Between-group comparisons were done with one-way ANOVAs for continuous variables, and Pearson chi-square tests for categorical variables. Post hoc, pairwise group comparisons were done in case of significant main effects of group.

#### Aim 1: ICV, IQ, and psychopathology

IQ and ICV were compared between groups using linear mixed-effects models with group as fixed effect (*α* = 0.05). As we included more than one offspring per family, family membership was added as random effect to account for similarities within families. Age and sex were added as covariates when comparing ICV between groups and analyses were repeated on subsets that were matched for age and sex. Post hoc, pairwise group comparisons were performed and Cohen’s *d* effect sizes were calculated. The lifetime prevalence of psychopathology based on the K-SADS-PL was compared between groups using Pearson chi-square tests (*α* = 0.05) and post hoc, pairwise group comparisons were done in case of significant main effects of group.

#### Aim 2: Group differences on global and local brain measures

Linear mixed-effects models were performed on brain measures, adding age, sex, and ICV as covariates (ICV was left out for mean/local cortical thickness and mean/local surface area). Local ROI volumes from the left and right hemisphere were summed to reduce the number of tests.

The significance level for the main effect of group for global brain volume analyses was set at *α* = 0.05. For subcortical volumes (*n* = 7) and cortical ROIs (*n* = 34), FDR correction for multiple testing was applied (*α* = 0.05; [41]). Post hoc pairwise comparisons are reported at an uncorrected *α* < 0.05 and effect sizes are reported.

#### Aim 3: Effect of psychopathology and IQ on ICV

Pearson correlations were calculated of IQ with ICV and whole brain volume in the total sample and in each group. Correlations were compared with Fisher-*r*-to-*z* transformation. IQ and the presence of psychiatric diagnosis (yes/no) were each added as an additional covariate in separate linear mixed models to investigate their effect on the group differences in brain measures.

Analyses were repeated, excluding subjects on medication (*N*_BDo_ = 4, *N*_SZo_ = 7). Analyses were conducted using Statistical Package for Social Sciences, version 23.0 (SPSS for Windows, IBM Corp, 2015) and R (https://www.R-project.org).

## Results

### Aim 1: ICV, IQ, and psychopathology

Table 1 provides an overview of the demographic and clinical characteristics of the groups. There was no significant difference between SZo and BDo in age, but BDo were significantly older than Co. In addition, there were significantly more females among SZo as compared to BDo.

**Table 1.** Offspring demographics, psychopathology and medication use, parental diagnoses, and parental education in schizophrenia offspring (SZo), bipolar offspring (BDo), and control offspring (Co)

Offspring characteristics	SZo (*N* = 40)	BDo (*N* = 66)	Co (*N* = 40)	Main effect		Pairwise		
						SZo vs. BDo	SZo vs. Co	BDo vs. Co
					*p*	*p*	*p*	*p*
Families	*N* = 30	*N* = 48	*N* = 30					
Sex m/f (% female)	12/28 (70%)	37/29 (44%)	21/19 (48%)	*X* ^2^ = 7.24	**0.03**	**0.01** (SZo > BDo)	**0.04** (SZo > Co)	0.72
Age *M* (*SD*)	13.77 (2.97)	14.52 (2.67)	12.74 (2.14)	*F* = 6.36	**0.002**	0.31	0.17	**0.001** (BDo > Co)
IQ *M* (*SD*)^a^	100.58 (19.17)	106.67 (18.30)	117.05 (13)	*F* = 9.11	**0.001**	0.10 *d* = 0.34	**<0.001** (SZo < Co) *d* = −1.06	**0.004** (BDo < Co) *d* = 0.67
Offspring lifetime axis I diagnoses *n* (%)^b^	SZo (*N* = 40)	BDo (*N* = 62)	Co (*N* = 39)					
Any diagnosis	22 (55%)	28 (45.2%)	7 (17.9%)	*X* ^2^ = 12.29	**0.002**	0.33	**0.001** (SZo > Co)	**0.01** (BDo > Co)
Any mood diagnosis	7 (17.5%)	17 (27.4%)	4 (10.3%)	*X* ^2^ = 4.63	0.10			
Major depression disorder	5 (12.5%)	1 (1.6%)	0	*X* ^2^ = 9.47	**0.01**	**0.02** (SZo > BDo)	**0.02** (SZo > Co)	0.43
Anxiety disorder	5 (12.5%)	4 (6.5%)	1 (2.6%)	*X* ^2^ = 3.03	0.22			
ADHD	2 (5%)	1 (1.6%)	0	*X* ^2^ = 2.51	0.29			
PDD/Asperger	5 (12.5%)	3 (4.8%)	0	*X* ^2^ = 5.91	0.05			
Other disorders	3 (7.5%)	3 (4.8%)	2 (5.1%)	*X* ^2^ = 0.35	0.84			
Medication use total sample (*n*)								
Antipsychotics	1	1	n.a.					
SSRI	1	1	n.a.					
Stimulants	4	1	n.a.					
Antipsychotics + SSRI	1	0	n.a.					
Stimulants + SSRI	0	1	n.a.					
Diagnosis index parent *n*								
Bipolar I disorder	n.a.	39	n.a.					
Bipolar II disorder	n.a.	8	n.a.					
Bipolar NOS		1						
Schizophrenia	19	n.a.	n.a.					
Schizoaffective disorder	7	n.a.	n.a.					
Psychosis NOS	4	n.a.	n.a.					
Highest level of parental education (% secondary/continued education)	38.7/61.3	28.6/71.4	19.4/80.6	*X* ^2^ = 3.04	0.22			

Abbreviations: ADHD, attention deficit hyperactivity disorder; BDo, bipolar disorder offspring; Co, control offspring; *M*, mean; n.a., not applicable; NOS, not otherwise specified; PDD, pervasive developmental disorder; *SD*, standard deviation; SSRI, selective serotonin reuptake inhibitor; SZo, schizophrenia offspring.

“Any mood diagnosis” also comprises major mood disorder; “other disorders” comprise eating disorder not otherwise specified, tic disorder, adjustment disorder with mixed disturbances of emotions and conduct, elimination disorder, and substance abuse disorder.

a IQ is missing in three BDo.

b For five participants, the administration of the interview did not coincide with the MRI scan date. The significance level was set at α = 0.05.

**Figure 1.**
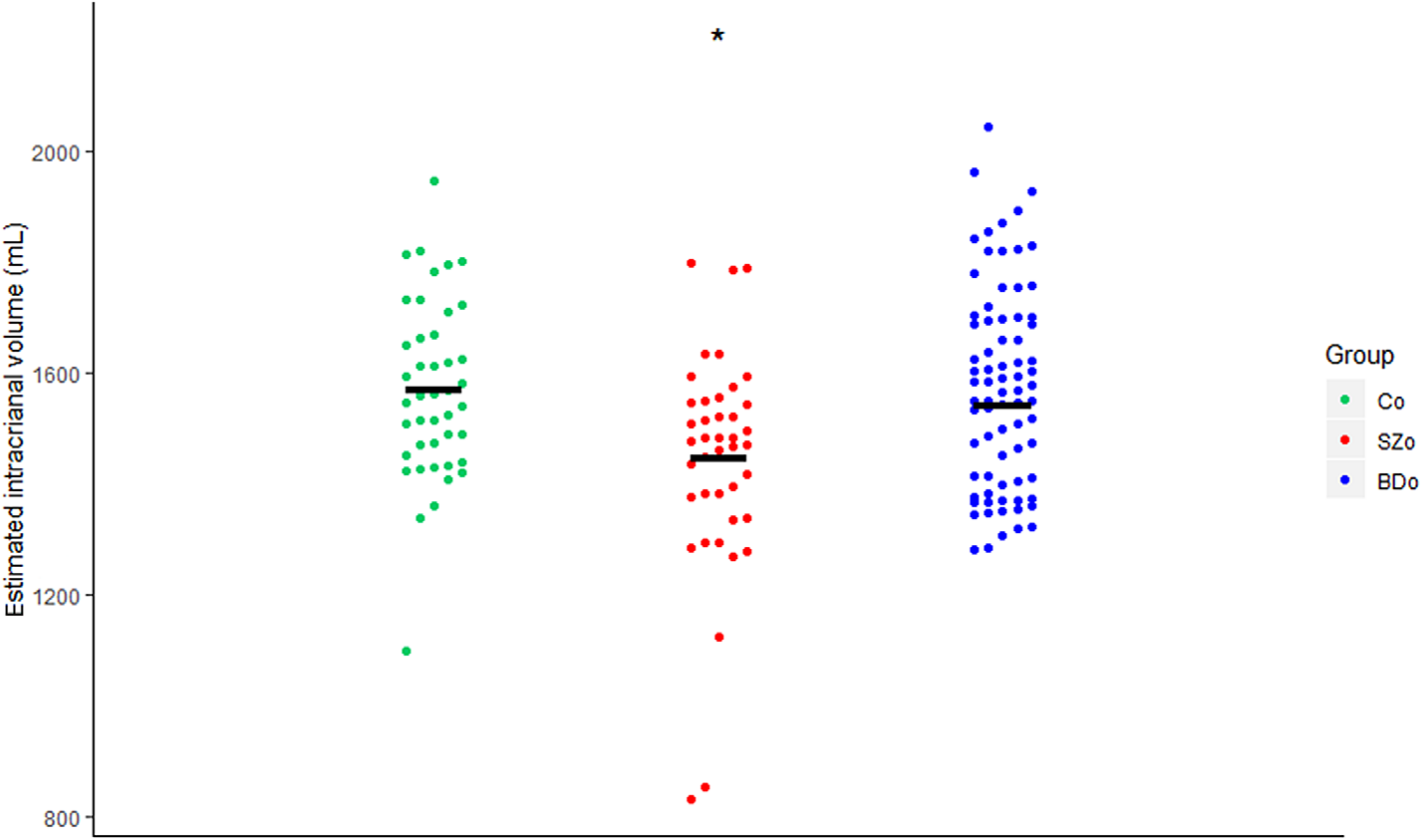
Estimated intracranial volume in SZo, BDo, and Co after correction for age and sex. Abbreviations: BDo, bipolar disorder offspring; Co, control offspring; SZo, schizophrenia offspring. SZo < BDo, *p* = 0.010; SZo < Co, *p* = 0.015.

A significant main effect of group was found for ICV. Pairwise comparisons revealed significantly smaller ICV in SZo than in BDo (*d* = −0.56) and Co (*d* = −0.59; Table 2/Figure 1). To match the subgroups on age, we excluded the 12 oldest BDo. This resulted in similar findings. To check if the smaller ICV in SZo was not explained by the lowest volumes, the analyses were repeated without three participants (all girls). Again, this resulted in similar findings (Table S4).

**Table 2.** Global brain volumes in schizophrenia offspring (SZo), bipolar offspring (BDo), and control offspring, corrected for age, sex, and intracranial volume

						Pairwise					
Global brain volumes	SZo (*N* = 40)	BDo (*N* = 66)	Co (*N* = 40)	Main effect		SZo < Co		BDo < Co		SZo < BDo	
	*M* (*SD*)	*M* (*SD*)	*M* (*SD*)	*F* (*df*, *df*)	*p*	*p*	*d* (CI 95%)	*p*	*d* (CI 95%)	*p*	*d* (CI 95%)
						Effect of group (corrected for age and sex)					
Intracranial (cm^3^)^a^	1,446 (200)	1,589 (183)	1,566 (161)	4.21 (2,107.8)	**0.017**	**0.015**	−0.59 (−1.03, −0.14)	0.917 (BDo > Co)	−0.03 (−0.42, 0.37)	**0.010**	−0.56 (−0.96, −0.16)
Cortical thickness (mm)^a^	2.6 (0.1)	2.6 (0.1)	2.7 (0.1)	4.67 (2,108.5)	**0.011**	**0.003**	−0.75 (−1.21, −0.3)	0.207	−0.28 (−0.67, 0.12)	**0.042**	−0.42 (−0.82, −0.03)
Cortical surface area (cm^2^)^a^	875 (98)	930 (93)	930 (84)	2.31 (2,108.8)	0.104	0.036	−0.52 (−0.96, −0.07)	0.384	−0.21 (−0.6, 0.19)	0.147	−0.32 (−0.71, 0.08)
						Effect of group (corrected for age, sex, and intracranial volume)					
Total brain (cm^3^)	1,127 (128)	1,208 (116)	1,225 (108)	2.74 (2,110.4)	0.069	0.067	−0.48 (−0.93, −0.04)	0.029	−0.53 (−0.93, −0.13)	0.891	0.03 (−0.36, 0.42)
Cortical GM (cm^3^)	526 (69)	560 (54)	578 (48)	2.18 (2,106.7)	0.118	0.051	−0.54 (−0.98, −0.09)	0.101	−0.38 (−0.78, 0.01)	0.588	−0.11 (−0.51, 0.28)
Subcortical GM (cm^3^)	63 (5)	67 (6)	67 (5)	1.24 (2,109.1)	0.293	0.124	−0.39 (−0.84, 0.05)	0.280	−0.24 (−0.63, 0.16)	0.525	−0.16 (−0.55, 0.23)
Cortical WM (cm^3^)	411 (53)	448 (60)	444 (54)	2.13 (2,112.6)	0.123	0.354	−0.26 (−0.7, 0.18)	0.042 (BDo > Co)	−0.46 (−0.86, −0.06)	0.319	0.22 (−0.17, 0.62)
Lateral ventricles (cm^3^)	11.6 (6.4)	15.6 (10.5)	11.3 (4)	3.55 (2,101.6)	**0.032**	0.090 (SZo > Co)	0.56 (0.12, 1.01)	**0.009** (BDo > Co)	0.55 (0.15, 0.95)	0.469	−0.15 (−0.54, 0.24)
Third ventricle (cm^3^)	0.8 (0.2)	0.8 (0.2)	07. (0.2)	2.32 (2,101.5)	0.104	0.039 (SZo > Co)	0.53 (0.09, 0.98)	0.480 (BDo > Co)	0.16 (−0.23, 0.56)	0.111 (SZo > BDo)	0.37 (−0.03, 0.76)
Cerebellum (cm^3^)	100 (13)	106 (10)	107 (11)	0.25 (2,109.1)	0.778	0.502	−0.15 (−0.59, 0.29)	0.858	−0.04 (−0.44, 0.35)	0.572	−0.13 (−0.53, 0.26)

Analyses are performed in a mixed model with correction for family membership.

Abbreviations: BDo, bipolar disorder offspring; Co, control offspring; d, Cohen’s d; df, degree of freedom; GM, gray matter; *M*, mean; *SD*, standard deviation; SZo, schizophrenia offspring; WM, white matter.

a Statistics for intracranial volume, mean cortical thickness and mean cortical surface area are not corrected for intracranial volume. The significance level was set at α = 0.05. FDR correction for multiple testing was applied for subcortical volumes and ROIs.

IQ was significantly lower in SZo (*d* = −1.06) and BDo (*d* = −0.67) as compared with Co. Although IQ was lower in SZo than in BDo, it did not differ significantly (*d* = −0.34).

SZo and BDo more often met criteria for a lifetime psychiatric disorder than Co. The frequency did not differ significantly between SZo and BDo. The types of psychopathology are presented in Table 1. No significant differences on age, sex, and IQ were found between those with and without a diagnosis (Table S1).

### Aim 2: Group differences on global and local brain measures

**Figure 2.**
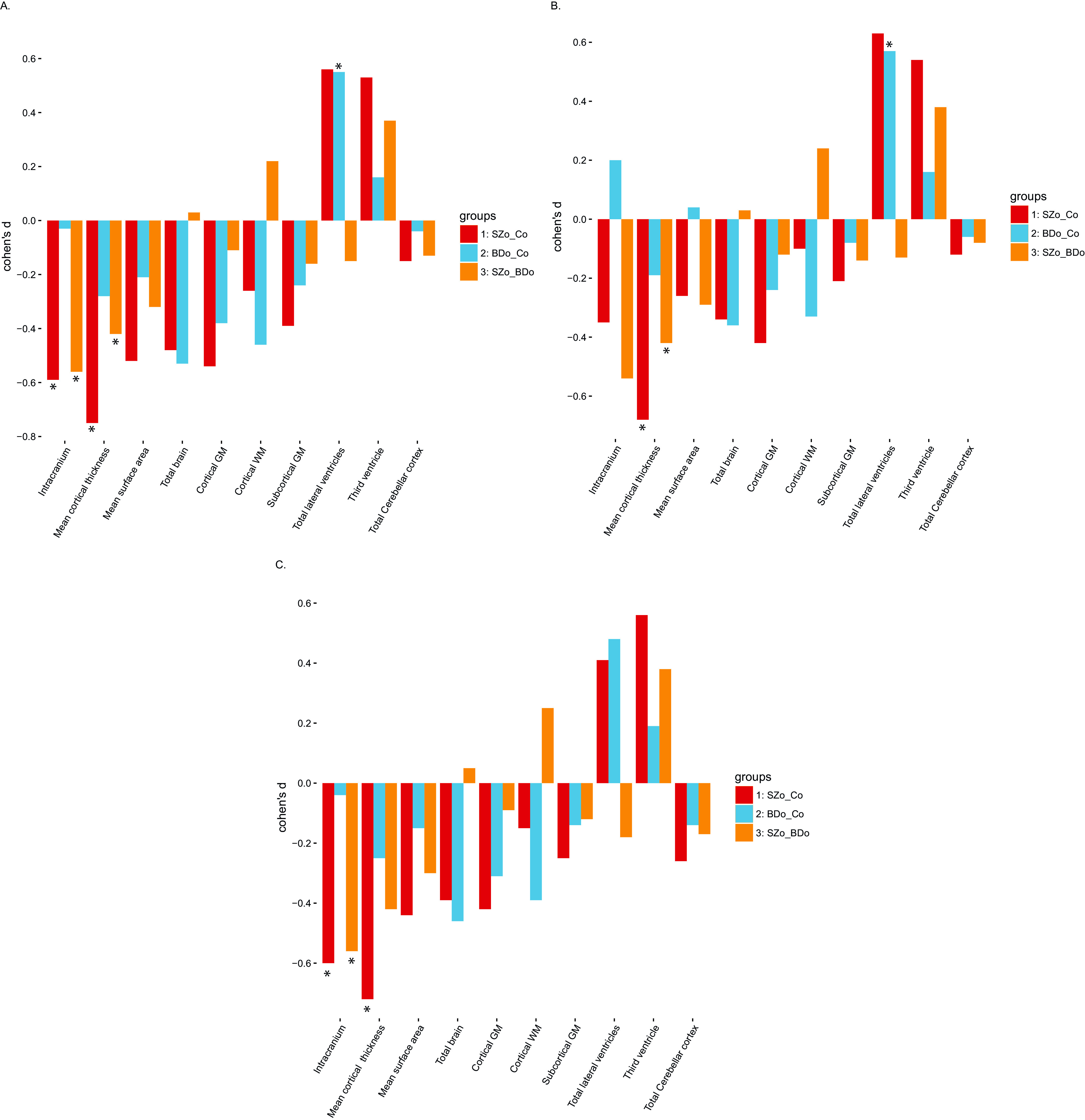
Effect sizes of pairwise comparison among SZo, BDo, and Co of global brain measures. (A) Corrected for age, sex, and ICV; (B) corrected for age, sex, ICV, and IQ; and (C) corrected for age, sex, ICV, and diagnosis. Abbreviations: BDo, bipolar disorder offspring; Co, control offspring; GM, gray matter; ICV, intracranial volume; SZo, schizophrenia offspring; WM, white matter.

Table 2 shows group differences in global brain measures, corrected for age, sex, and ICV. The cortex was significantly thinner in SZo than in BDo (*d* = −0.42) and Co (*d* = −0.75) and lateral ventricle volume was significantly larger in BDo than in Co (*d* = 0.55). Surprisingly, the effect size of the SZo–Co comparison of lateral ventricle volume (*d* = 0.56) was comparable to that of the BDo–Co comparison; however, the first did not reach significance (*p* = 0.09) while the latter did. Therefore, we cannot rule out that the enlargement in lateral ventricle volume is not specific to BDo. Effect sizes of pairwise comparisons of global brain measures among SZo, BDo, and Co are presented in Figure 2. Subcortical and local cortical volumes were not significantly different between groups after correction for age, sex, and ICV (Table S2).

### Aim 3: Effect of psychopathology and IQ on ICV

ICV and whole brain (WB) volume correlated significantly with IQ across all subjects (*r*_ICV-IQ_ = 0.37, *p* = 0.7 × 10^−5^ and *r*_WB-IQ_ = 0.47, *p* = 0.4 × 10^−8^) and correlations were comparable between groups, albeit non-significant in Co for ICV (ICV-IQ: *r*_SZo_ = 0.32, *p* = 0.05; *r*_BDo_ = 0.41, *p* = 0.001; *r*_Co_ = 0.21, *p* = 0.20; WB-IQ: *r*_SZo_ = 0.53, *p* < .001; *r*_BDo_ = 0.39, *p* = 0.002; *r*_Co_ = 0.34, *p* = 0.03). Using Fisher *r*-to-*z* transformation, pairwise differences between these correlations were not significant (all *p* > 0.21).

After IQ correction, the main effect of group on ICV remained trend-level significant (*p* = 0.05). Pairwise comparisons showed that ICV remained smaller in SZo compared to BDo with a comparable effect size (*d* = −0.54); however, the difference between SZo and Co was no longer significant and had a moderately smaller effect size (*d* = −0.35; Table S3).

After correction for the presence of a psychiatric diagnosis, the main effect of group on ICV remained significant (*p* = 0.03) and pairwise analyses showed that ICV is smaller in SZo compared to Co (*d* = −0.60) and BDo (*d* = −0.56). The findings in the global and local brain measures after correction for IQ or presence of a psychiatric diagnosis remained similar (Table S3).

Finally, repeating the main analyses without the 11 offspring who used psychotropic medication did not change the pattern of findings (results are available upon request).

## Discussion

The current study aimed to investigate whether early neurodevelopment, as expressed by ICV, is differentially affected in young SZo and BDo. Our main finding is that SZo had a significantly smaller ICV than BDo and Co. This corresponds with the smaller ICV found in adult SZ patients, but not in BD patients, compared to controls [9]. Thus far, studies on ICV of SZo and BDo are sparse and findings are inconsistent [27,29,42]. Sugranyes et al. [42] showed no significant differences in ICV between SZo (*n* = 38), BDo (*n* = 77), and Co (*n* = 83), aged 6–17 years. Other studies, focusing on one disorder, reported decreased ICV in SZo (*n* = 17) compared to Co (*n* = 22), aged 9–22 years [29] and no significant differences between BDo (*n* = 26) and Co (*n* = 31), aged 15–30 years [27]. These and our findings suggest that a smaller ICV may be specific for SZo. The question remains, at what critical stage during development the ICV stays behind in SZo. It has been reported that the first 5 years of life are characterized by rapid linear growth of ICV, with another spurt between 10 and 15 years of age [4]. Additionally, head circumference (i.e., a proxy for ICV) was smaller in babies who later developed SZ [43,44]. Further evidence suggests a lower height at age two in children who later developed a nonaffective psychotic disorder [45]. These studies suggest that prenatal risk factors or diminished growth during the first 5 years may explain our finding of a smaller ICV in child and adolescent SZo. Whether SZo and BDo differ in ICV-growth during adolescence remains unclear. Longitudinal studies in large samples including children and adolescents between 8 and 20 years old are needed to address this question.

Importantly, IQ was significantly lower and the prevalence of psychiatric disorders was significantly higher in both SZo and BDo as compared to Co. With respect to SZ, level of IQ has also been considered an indirect indication of aberrant neurodevelopment, as IQ-scores are below those of healthy subjects, years before diagnosis [46–51]. In contrast, recent evidence suggests that a U-shaped effect explains the relationship between IQ and risk for BD, with an even higher risk for above-average cognitive performance than low IQ [52,53]. However, in our cohort, both SZo and BDo had a lower IQ than controls. Moreover, we found no differences in IQ between offspring with and without a DSM-IV diagnosis.

The lower IQ and higher burden of psychopathology did not fully explain the smaller ICV in SZo. After IQ correction, ICV was still smaller in SZo than in BDo with a similar effect size, but the difference between SZo and Co was no longer significant. Correcting for the presence of a psychiatric diagnosis did not affect the ICV findings. This suggests that it is not the presence of psychopathology, but the increased familial risk for SZ that influences brain development in early life leading to a smaller ICV. Taken together, our finding of a smaller ICV in SZo than in BDo provides evidence for aberrant (possibly early) brain development in relation to SZ risk, which is in line with the neurodevelopmental model of SZ [54,55]. Future studies with larger samples may have increased statistical power to address the possible differential associations between IQ and ICV with familial risk for SZ or BD.

In addition to ICV, we compared the groups on other brain measures after correcting for ICV. We found a significantly thinner cortex in SZo than in BDo and Co and larger lateral ventricle volume in BDo than in Co. The SZo-Co comparison of lateral ventricle volume was not statistically significant, despite a comparable effect size. The absence of significant differences in local cortical ROI volumes suggests that brain abnormalities in relation to increased familial risk for BD or SZ represent mostly a global, and not a local effect. Alternatively, the sample size may have been too small to identify subtle local abnormalities in SZo and BDo. Whether local brain abnormalities play a role in the prediction of future illness onset remains to be elucidated when longitudinal data become available.

Our local brain imaging findings differ from those of a Spanish SZo/BDo cohort, who reported smaller local volumes in the left inferior frontal gyrus/anterior insula in SZo relative to both BDo and Co. In addition, they did not find a significant difference in ICV, but found a smaller surface area in SZo than in BDo and Co [42,56]. ICV and surface area are usually highly correlated [57], suggesting that the smaller ICV in our study and the smaller surface area in the Spanish cohort in SZo may represent the same underlying process. Other offspring studies reported smaller volumes of the whole brain, frontal and temporal lobes, corpus callosum, and subcortical structures in SZo compared to Co, while brain abnormalities in BDo are less conclusive [25–29,31–34,58]. Taken together, brain abnormalities appear more pronounced in SZo compared to BDo.

Despite our relatively large sample size and the inclusion of both SZo and BDo at one site (with one scanner), some limitations have to be considered. SZo were more often referred by their treating psychiatrist than BDo, who were more often referred by the parent’s physician. It is possible that this may have resulted in more severe levels of psychopathology in SZo and we cannot rule out that this influenced our results. In addition, controls had a rather high level of IQ, although our groups were matched on parental education. Finally, the current study presents cross-sectional data, which does not allow to differentiate between deficits in neurodevelopment and a delay in neurodevelopment. However, when longitudinal data becomes available, it will be possible to investigate the neurodevelopmental trajectories toward specific disorders, taking into account other early developmental and environmental factors (e.g., circumstances of gestation, birth and upbringing, and cumulative life events).

To conclude, the familial risk for SZ may be related to stunted brain development early in life, which is less for BD. Irrespective of a lower IQ and increased presence of psychopathology in SZo and BDo, abnormal early neurodevelopment (expressed by a smaller ICV) differentiated SZo from BDo and Co. In addition, the cortex was thinner in SZo and lateral ventricles were larger in BDo (and possibly also in SZo), confirming that brain abnormalities appear more pronounced in SZo than in BDo. The lack of local brain volume abnormalities in the familial high-risk groups may suggest that brain abnormalities related to the risk to develop SMI are related to processes that affect global brain measures, but larger samples are needed to confirm this.

## Financial Support

This study was supported by Brain and Behavior Research Foundation (2013–2015 NARSAD Independent Investigator grant number 20244 to M.H.J.H.); and The Netherlands Organization for Scientific Research (2012–2017 VIDI grant number 452–11-014 to N.E.M.H.).

## Conflict of Interest

The authors declare no conflict of interests.

## Supplementary material

For supplementary material accompanying this paper visit https://doi.org/10.1192/j.eurpsy.2019.19. click here to view supplementary material
