# Supplementary material for: Brain structure, IQ, and psychopathology in young offspring of patients with schizophrenia or bipolar disorder
Source: Eur Psychiatry. 2020 Jan 31;63(1):e5. doi: 10.1192/j.eurpsy.2019.19 (PMC8057400; doi:10.1192/j.eurpsy.2019.19)
Supplement: Supplementary file 1 [file epasup.zip › S0924933819000191sup001.docx]

|  | | | | |
| --- | --- | --- | --- | --- |
| **Table S1. Age, sex and IQ in SZo, BDo, and Co, comparing offspring with and without diagnosis** | | | | |
|  | **Diag+** | **Diag-** |  | |
| **N** |  |  |  |  |
| SZo | 22 | 18 |  |  |
| BDo | 28 | 34 |  |  |
| Co | 7 | 32 |  |  |
|  |  |  |  |  |
| **Age *M (SD)*** |  |  |  | *p* |
| SZo | 14.22 (3.21) | 13.03 (2.60) | *t* = 1.26 | 0.21 |
| BDo | 15.01 (2.47) | 14.56 (2.93) | *t* = 0.64 | 0.52 |
| Co | 12.67 (2.23) | 12.77 (2.18) | *t* = -0.11 | 0.92 |
| **sex male/female (% female)** |  |  |  | *p* |
| SZo | 9/13 (59.1%) | 3/15 (83.3%) | *X^2^* = 2.77 | 0.10 |
| BDo | 18/10 (35.7%) | 18/16 (47.1%) | *X^2^* = 0.81 | 0.37 |
| Co | 3/4 (57.1%) | 18/14 (43.8%) | *X^2^* = 0.42 | 0.52 |
| **IQ *M (SD)*** |  |  |  | *p* |
| SZo | 99.27 (19.18) | 102.17 (19.58) | *t* = -0.47 | 0.64 |
| BDo | 105.59 (18.72) | 107.43 (17.74) | *t* = -0.39 | 0.70 |
| Co | 118.43 (13.14) | 116.38 (13.18) | *t* = 0.37 | 0.71 |
| Abbreviations: BDo, bipolar disorder offspring; Co, Control offspring; Diag+, offspring with diagnosis; Diag-, offspring without diagnosis; *M,*  mean; *SD,* standard deviation; SZo, schizophrenia offspring. The significance level was set at α = 0.05. | | | | |

| **Table S2. Subcortical and cortical ROI volumes in schizophrenia offspring (SZo), bipolar disorder offspring (BDo) and control offspring, corrected for age, sex and intracranial volume** | | | | | | | | |
| --- | --- | --- | --- | --- | --- | --- | --- | --- |
|  | Main effect | | Pairwise | | | | | |
| **Subcortical volumes** |  | FDR  (α=0.05)  =NS | *SZo < Co* |  | *BDo < Co* |  | *SZo < BDo* |  |
|  | *F (df, df)* | *p* | *p* | *d* (CI 95%) | *p* | *d* (CI 95%) | *p* | *d* (CI 95%) |
|  |  |  | **Effect of GROUP**  **(corrected for age, sex, and intracranial volume)** | | | | | |
| Thalamus (cmᶟ) | 0.29 (2, 110.1) | 0.752 | 0.728 | -0.09  (-0.52, 0.35) | 0.453  (BDo > Co) | -0.16  (-0.56, 0.23) | 0.721 | 0.09  (-0.30, 0.48) |
| Hippocampus (cmᶟ) | 2.54 (2, 106.7) | 0.083 | 0.233 | -0.30  (-0.74, 0.14) | 0.026  (BDo > Co) | -0.50  (-0.9, -0.1) | 0.369  *(SZo > BDo)* | 0.22  (-0.18, 0.61) |
| Amygdala (cmᶟ) | 0.61 (2, 105.1) | 0.607 | 0.284 | -0.30  (-0.74, 0.14) | 0.695 | -0.09  (-0.48, 0.31) | 0.426 | -0.18  (-0.58, 0.21) |
| Caudate (cmᶟ) | 1.09 (2, 114.1) | 0.341 | 0.838 | 0.05  (-0.38, 0.49) | 0.185 | 0.29  (-0.1, 0.68) | 0.278 | -0.25  (-0.65, 0.14) |
| Putamen (cmᶟ) | 0.78 (2, 111.4) | 0.460 | 0.217 | -0.37  (-0.81, 0.07) | 0.590 | -0.12  (-0.51, 0.27) | 0.406 | -0.19  (-0.58, 0.21) |
| Pallidum (cmᶟ) | 0.79 (2, 89.7) | 0.457 | 0.254 | -0.31  (-0.75, 0.13) | 0.840 | -0.04  (-0.44, 0.35) | 0.288 | -0.24  (-0.63, 0.15) |
| Accumbens (cmᶟ) | 2.77 (2,97.1) | 0.068 | 0.030 | -0.56  (-1.01, -0.11) | 0.061 | -0.43  (-0.82, -0.03) | 0.571 | -0.14  (-0.53, 0.25) |
| **ROI cortical volume** |  | FDR  (α=0.05)  =NS |  |  |  |  |  |  |
| Banks of superior temporal (cmᶟ) | 2.22 (2,107.6) | 0.114 | 0.061 | -0.49  (-0.94, -0.05) | 0.789 | -0.06  (-0.45, 0.33) | 0.070 | -0.40  (-0.79, 0.00) |
| Caudal anterior cingulate (cmᶟ) | 0.31 (2,104.4) | 0.736 | 0.505 | -0.16  (-0.6, 0.28) | 0.480 | -0.16  (-0.55, 0.24) | 0.970 | -0.01  (-0.40, 0.38) |
| Caudal middle frontal (cmᶟ) | 0.09 (2,110.3) | 0.911 | 0.904 | -0.03  (-0.47, 0.41) | 0.779  (BDo > Co) | 0.06  (-0.33, 0.46) | 0.682 | -0.09  (-0.48, 0.30) |
| Cuneus (cmᶟ) | 1.02 (2,140.0) | 0.364 | 0.180 | -0.31  (-0.75, 0.13) | 0.261 | -0.25  (-0.65, 0.14) | 0.713 | -0.08  (-0.47, 0.32) |
| Entorhinal (cmᶟ) | 1.23 (2,99.1) | 0.297 | 0.121 | -0.38  (-0.82, 0.06) | 0.446 | -0.16  (-0.55, 0.23) | 0.336 | -0.21  (-0.61, 0.18) |
| Fusiform (cmᶟ) | 0.49 (2,112.3) | 0.617 | 0.962 | -0.01  (-0.45, 0.42) | 0.427  (BDo > Co) | 0.17  (-0.22, 0.56) | 0.402 | -0.18  (-0.57, 0.21) |
| Inferior parietal (cmᶟ) | 0.50 (2,88.7) | 0.606 | 0.344 | -0.24  (-0.68, 0.2) | 0.432 | -0.17  (-0.56, 0.22) | 0.789 | -0.05  (-0.45, 0.34) |
| Inferior temporal (cmᶟ) | 0.53 (2,108.7) | 0.589 | 0.311 | -0.26  (-0.7, 0.18) | 0.490 | -0.15  (-0.55, 0.24) | 0.660 | -0.10  (-0.49, 0.29) |
| Insthmus cingulate (cmᶟ) | 1.79 (2,110.8) | 0.171 | 0.793 | -0.07  (-0.51, 0.37) | 0.090 | -0.38  (-0.78, 0.02) | 0.164 | 0.32  (-0.08, 0.71) |
| Lateral occipital (cmᶟ) | 3.21 (2,105.8) | 0.044 | 0.022 | -0.56  (-1, -0.11) | 0.036 | -0.48  (-0.88, -0.08) | 0.640 | -0.11  (-0.50, 0.29) |
| Lateral orbitofrontal (cmᶟ) | 0.21 (2,107.7) | 0.808 | 0.656 | -0.12  (-0.55, 0.32) | 0.521 | -0.15  (-0.54, 0.24) | 0.887 | 0.03  (-0.36, 0.42) |
| Lingual (cmᶟ) | 1.88 (2,105.1) | 0.158 | 0.157 | -0.37  (-0.81, 0.08) | 0.063 | -0.42  (-0.82, -0.03) | 0.779 | 0.06  (-0.33, 0.45) |
| Medial orbito frontal (cmᶟ) | 0.13 (2,101.5) | 0.876 | 0.619 | -0.12  (-0.56, 0.32) | 0.705 | -0.09  (-0.48, 0.3) | 0.861 | -0.04  (-0.43, 0.35) |
| Middle temporal (cmᶟ) | 0.13 (2,101.9) | 0.129 | 0.052 | -0.57  (-1.02, -0.12) | 0.537 | -0.13  (-0.52, 0.26) | 0.122 | -0.34  (-0.74, 0.06) |
| Parahippocampal (cmᶟ) | 0.98 (2,111.2) | 0.379 | 0.797 | -0.07  (-0.51, 0.37) | 0.200 | -0.28  (-0.68, 0.11) | 0.324 | 0.23  (-0.17, 0.62) |
| Paracental (cmᶟ) | 3.08 (2,88.6) | 0.051 | 0.035 | -0.51  (-0.96, -0.07) | 0.028 | -0.49  (-0.88, -0.09) | 0.877 | -0.04  (-0.43, 0.36) |
| Pars opercularis (cmᶟ) | 0.05 (2,88.4) | 0.948 | 0.945 | -0.02  (-0.46, 0.42) | 0.818 | 0.05  (-0.34, 0.44) | 0.761 | -0.06  (-0.46, 0.33) |
| Pars orbitalis (cmᶟ) | 3.03 (2,114.3) | 0.052 | 0.045 | -0.52  (-0.97, -0.08) | 0.024 | -0.51  (-0.91, -0.11) | 0.988 | 0.00  (-0.39, 0.40) |
| Pars triangularis (cmᶟ) | 1.54 (2,109.2) | 0.218 | 0.114 | -0.42  (-0.86, 0.03) | 0.138 | -0.31  (-0.7, 0.09) | 0.778 | -0.06  (-0.45, 0.33) |
| Pericalcarine (cmᶟ) | 0.46 (2,103.9) | 0.630 | 0.948 | 0.02  (-0.42, 0.45) | 0.444 | -0.19  (-0.58, 0.2) | 0.408 | 0.19  (-0.21, 0.58) |
| Postcentral (cmᶟ) | 4.94 (2,140.0) | 0.008 | 0.014 | -0.57  (-1.01, -0.12) | 0.004 | -0.66  (-1.06, -0.25) | 0.852 | 0.04  (-0.35, 0.43) |
| Posterior cingulate (cmᶟ) | 2.40 (2,91.4) | 0.097 | 0.459 | -0.19  (-0.63, 0.25) | 0.036 | -0.49  (-0.89, -0.09) | 0.202 | 0.27  (-0.13, 0.66) |
| Precental (cmᶟ) | 0.83 (2,103.1) | 0.440 | 0.413 | -0.20  (-0.64, 0.24) | 0.204 | -0.30  (-0.69, 0.1) | 0.723 | 0.08  (-0.31, 0.47) |
| Precuneus (cmᶟ) | 2.56 (2,140.0) | 0.081 | 0.116 | -0.40  (-0.85, 0.04) | 0.028 | -0.48  (-0.87, -0.08) | 0.650 | 0.09  (-0.30, 0.49) |
| Rostral anterior cingulate (cmᶟ) | 0.48 (2,98.3) | 0.620 | 0.394 | -0.22  (-0.66, 0.22) | 0.944 | -0.02  (-0.41, 0.38) | 0.383 | -0.20  (-0.59, 0.20) |
| Rostral middle frontal (cmᶟ) | 0.45 (2,108.5) | 0.637 | 0.347 | -0.24  (-0.68, 0.19) | 0.538 | -0.15  (-0.54, 0.25) | 0.667 | -0.09  (-0.49, 0.30) |
| Superior frontal (cmᶟ) | 1.41 (2,99.1) | 0.249 | 0.104 | -0.39  (-0.83, 0.06) | 0.230 | -0.29  (-0.68, 0.11) | 0.540 | -0.14  (-0.53, 0.26) |
| Superior parietal (cmᶟ) | 0.07 (2,99.4) | 0.930 | 0.764 | -0.08  (-0.52, 0.36) | 0.720 | -0.08  (-0.48, 0.31) | 0.982 | 0.01  (-0.39, 0.40) |
| Superior temporal (cmᶟ) | 2.42 (2,112.1) | 0.094 | 0.035 | -0.56  (-1.01, -0.11) | 0.108 | -0.36  (-0.75, 0.04) | 0.451 | -0.16  (-0.56, 0.23) |
| Supramarginal (cmᶟ) | 3.05 (2,102.5) | 0.052 | 0.015 | -0.59  (-1.04, -0.15) | 0.174 | -0.32  (-0.71, 0.08) | 0.172 | -0.29  (-0.68, 0.11) |
| Frontal pole (cmᶟ) | 1.00 (2,104.8) | 0.370 | 0.525 | -0.15  (-0.59, 0.29) | 0.481 | 0.16  (-0.23, 0.56) | 0.163 | -0.33  (-0.73, 0.06) |
| Temporal pole (cmᶟ) | 1.16 (2,105.7) | 0.318 | 0.134 | -0.37  (-0.81, 0.07) | 0.324 | -0.21  (-0.6, 0.18) | 0.493 | -0.15  (-0.55, 0.24) |
| Transverse temporal (cmᶟ) | 2.42 (2,98.3) | 0.094 | 0.033 | -0.55  (-1, -0.1) | 0.126 | -0.35  (-0.75, 0.04) | 0.394 | -0.20  (-0.60, 0.19) |
| Insula (cmᶟ) | 4.77 (2,104.7) | 0.010 | 0.006 | -0.73  (-1.18, -0.27) | 0.011 | -0.57  (-0.97, -0.17) | 0.594 | -0.12  (-0.51, 0.28) |
| Analyses are performed in a mixed model with correction for family membership. Abbreviations: BDo, bipolar disorder offspring; CI, confidence interval; Co, control offspring ; d, Cohen’s d; df, degree of freedom; FDR, false discovery rate; GM, gray matter; SZo, schizophrenia offspring; WM, white matter. ^a^ Statistics for intracranial volume, mean cortical thickness and mean cortical surface area are not corrected for intracranial volume. The significance level was set at α= 0.05. | | | | | | | | |

| **Table S3. (A) Measures of global brain volume, mean cortical thickness, mean cortical surface area, (B) subcortical, and (C) cortical brain volumes in schizophrenia offspring (SZo), bipolar offspring (BDo) and controls (Co) after correction for age, sex, ICV and IQ, and after correction for age, sex, ICV and diagnosis.** | | | | | | | | | | | | | | | | | |
| --- | --- | --- | --- | --- | --- | --- | --- | --- | --- | --- | --- | --- | --- | --- | --- | --- | --- |
| **(A) Global brain volumes** | Main effect | | Pairwise | | | | | | | Main effect | | Pairwise | | | | | |
|  |  |  | SZo < Co | | BDo < Co | | | SZo < BDo | |  |  | SZo < Co | | BDo < Co | | SZo < BDo | |
|  | *F (df,df)* | *p* | *p* | *d* (CI 95%) | *p* | *d* (CI 95%) | | *p* | *d* (CI 95%) | *F (df,df)* | *p* | *p* | *d* (CI 95%) | *p* | *d* (CI 95%) | *p* | *d* (CI 95%) |
|  |  |  | **Effect of GROUP**  **(corrected for age, sex and IQ)** | | | | | | |  |  | **Effect of GROUP**  **(corrected for age, sex and diagnosis)** | | | | | |
| Intracranial volume | 3.07  (2,101.54) | 0.05 | 0.16 | -0.35  (-0.79 - 0.09) | 0.41 | 0.2  (-0.19, 0.6) | | 0.02 | -0.54  (-0.94, -0.14) | 3.82  (2,107.46) | **0.03** | 0.03 | -0.6  (-1.05, -0.15) | 0.89 | -0.04  (-0.43, 0.36) | 0.01 | -0.56  (-0.96, -0.16) |
| Cortical thickness (mm) | 3.50  (2,104.39) | **0.03** | 0.01 | -0.68  (-1.13, -0.23) | 0.39 | -0.19  (-0.59, 0.2) | | 0.05 | -0.42  (-0.82, -0.02 ) | 3.49  (2,106.26) | **0.03** | 0.01 | -0.72  (-1.17, -0.27) | 0.28 | -0.25  (-0.65, 0.14) | 0.05 | -0.42  (-0.81, -0.02) |
| Cortical surface area (cm2) | 0.93  (2,101.46) | 0.40 | 0.32 | -0.26  (-0.7, 0.18) | 0.86 | 0.04  (-0.35, 0.44) | | 0.19 | -0.29  (-0.69, 0.11) | 1.46  (2,108.30) | 0.24 | 0.11 | -0.44  (-0.89, 0) | 0.56 | -0.15  (-0.55, 0.24) | 0.18 | -0.3  (-0.7, 0.09) |
|  |  |  | **Effect of GROUP**  **(corrected for age, sex, ICV and IQ)** | | | | | | |  |  | **Effect of GROUP**  **(corrected for age, seks, ICV and diagnosis)** | | | | | |
| Total brain (cmᶟ) | 1.22  (2,105.15) | 0.30 | 0.23 | -0.34  (-0.78, 0.1) | 0.14 | | -0.36  (-0.76, 0.04) | 0.88 | 0.03  (-0.36, 0.43) | 1.63  (2,111.68) | 0.20 | 0.18 | -0.39  (-0.83, 0.05) | 0.08 | -0.46  (-0.86, -0.07) | 0.81 | 0.05  (-0.34, 0.45) |
| Cortical GM (cmᶟ) | 1.07  (2,102.31) | 0.35 | 0.15 | -0.42  (-0.87, 0.02) | 0.30 | | -0.24  (-0.64, 0.16) | 0.56 | -0.12  (-0.52, 0.28 ) | 1.07  (2,104.12) | 0.35 | 0.17 | -0.42  (-0.87, 0.02) | 0.22 | -0.31  (-0.71, 0.08) | 0.68 | -0.09  (-0.48, 0.31) |
| Subcortical GM (cmᶟ) | 0.32  (2,106.80) | 0.72 | 0.43 | -0.21  (-0.65, 0.23) | 0.73 | | -0.08  (-0.47, 0.32) | 0.59 | -0.14  (-0.53, 0.26 ) | 0.386  (2,110.12) | 0.68 | 0.38 | -0.25  (-0.69, 0.19) | 0.56 | -0.14  (-0.53, 0.25) | 0.65 | -0.12  (-0.51, 0.28) |
| Cortical white matter (cmᶟ) | 1.16  (2,109.47) | 0.32 | 0.75 | -0.1  (-0.54, 0.34) | 0.16 | | -0.33  (-0.73, 0.07) | 0.29 | 0.24  (-0.15, 0.64) | 1.52  (2,115.05) | 0.22 | 0.63 | -0.15  (-0.59, 0.29) | 0.11 | -0.39  (-0.79 , 0) | 0.27 | 0.25  (-0.14. 0.65) |
| Lateral ventricles (cmᶟ) | 3.59  (2,98.34) | **0.03** | 0.07 | 0.63  (0.18, 1.08) | 0.01 | | 0.57  (0.17, 0.98) | 0.53 | -0.13  (-0.53, 0.26) | 2,26  (2,100.37) | 0.11 | 0.27 | 0.41  (-0.03, 0.86) | 0.04 | 0.48  (0.08, 0.88) | 0.39 | -0.18  (-0.58, 0.21) |
| 3rd ventricle (cmᶟ) | 2,28  (2,99.20) | 0.11 | 0.04 | 0.54  (0.1, 0.99) | 0.51 | | 0.16  (-0.24, 0.55) | 0.11 | 0.38  (-0.02, 0.78) | 2,13  (2,101.00) | 0.12 | 0.05 | 0.56  (0.12, 1.01) | 0.45 | 0.19  (-0.21, 0.58) | 0.11 | 0.38  (-0.02, 0.77) |
| Cerebellum (cmᶟ) | 0.13  (2,103.50) | 0.88 | 0.62 | -0.12  (-0.56, 0.32) | 0.80 | | -0.06  (-0.46, 0.33) | 0.76 | -0.08  (-0.47, 0.32) | 0.52  (2,108.71) | 0.59 | 0.31 | -0.26  (-0.7, 0.18) | 0.61 | -0.14  (-0.53, 0.26) | 0.49 | -0.17  (-0.56, 0.23) |
| **(B) Subcortical volumes** |  | FDR  (α=0.05)  =NS |  | |  | | |  | |  | FDR  (α=0.05)  =NS |  | |  | |  | |
| Thalamus (cmᶟ) | 0.17  (2,107.65) | 0.84 | 0.78 | 0.07  (-0.37, 0.51) | 0.79 | | -0.06  (-0.46, 0.34) | 0.56 | 0.15  (-0.25, 0.54) | 0.19  (2,110.67) | 0.83 | 0.88 | -0.04  (-0.48, 0.4) | 0.57 | -0.13  (-0.53, 0.26) | 0.68 | 0.1  (-0.29, 0.5) |
| Hippocampus (cmᶟ) | 1.91  (2,103.35) | 0.15 | 0.31 | -0.27  (-0.71, 0.17) | 0.05 | | -0.45  (-0.85, -0.05) | 0.41 | 0.2  (-0.2, 0.6) | 1.77  (2,105.26) | 0.18 | 0.46 | -0.21  (-0.65, 0.23) | 0.07 | -0.43  (-0.83, -0.04) | 0.32 | 0.24  (-0.15, 0.64) |
| Amygdala (cmᶟ) | 0.31  (2,102,04) | 0.74 | 0.56 | -0.16  (-0.6, 0.28) | 0.91 | | 0.02  (-0.37, 0.42) | 0.45 | -0.18  (-0.58, 0.22) | 0.15  (2,100.11) | 0.86 | 0.88 | -0.05  (-0.49, 0.39) | 0.75 | 0.07  (-0.32, 0.47) | 0.60 | -0.12  (-0.52, 0.27) |
| Caudate (cmᶟ) | 1.71  (2,111.76) | 0.19 | 0.43 | 0.22  (-0.22, 0.66) | 0.07 | | 0.42  (0.01, 0.82) | 0.35 | -0.22  (-0.62, 0.18) | 1.34  (2,114.10) | 0.27 | 0.57 | 0.16  (-0.27, 0.6) | 0.12 | 0.37  (-0.03, 0.77) | 0.35 | -0.22  (-0.62, 0.17) |
| Putamen (cmᶟ) | 0.46  (2,109.08) | 0.63 | 0.42 | -0.25  (-0.69, 0.19) | 0.97 | | -0.01  (-0.4, 0.39) | 0.38 | -0.2  (-0.59, 0.2) | 0.26  (2,113.93) | 0.77 | 0.54 | -0.2  (-0.64, 0.24) | 0.93 | -0.02  (-0.41, 0.37) | 0.51 | -0.15  (-0.54, 0.24) |
| Pallidum (cmᶟ) | 0.59  (2,87.71) | 0.55 | 0.37 | -0.25  (-0.69, 0.19) | 1.00 | | 0  (-0.39, 0.4) | 0.31 | -0.23  (-0.63, 0.17) | 0.89  (2,88.72) | 0.42 | 0.22 | -0.38  (-0.82, 0.07) | 0.72 | -0.08  (-0.48, 0.31) | 0.26 | -0.26  (-0.65, 0.14) |
| Accumbens (cmᶟ) | 1.89  (2,93.44) | 0.16 | 0.07 | -0.49  (-0.94, -0.05) | 0.13 | | -0.35  (-0.75, 0.05) | 0.57 | -0.14  (-0.54, 0.25) | 2,39  (2,98.22) | 0.10 | 0.04 | -0.59  (-1.04, -0.15) | 0.07 | -0.45  (-0.85, -0.05) | 0.55 | -0.15  (-0.54, 0.24) |
| **(C) ROI cortical volume** |  | FDR  (α=0.05)  =0.015 |  |  |  | |  |  |  |  | FDR  (α=0.05) =  0.015 |  |  |  |  |  |  |
| Banks of superior temporal (cmᶟ) | 1.94  (2,100.96) | 0.15 | 0.11 | -0.43  (-0.88, 0.01) | 0.96 | | 0.01  (-0.38, 0.41) | 0.07 | -0.4  (-0.8, 0) | 1.735  (2,104.47) | 0.18 | 0.13 | -0.45  (-0.89, 0) | 0.90 | -0.03  (-0.42, 0.36) | 0.08 | -0.38  (-0.78, 0.01) |
| Caudal anterior cingulate (cmᶟ) | 0.26  (2,99.70) | 0.77 | 0.61 | -0.13  (-0.57, 0.31) | 0.48 | | -0.16  (-0.56, 0.24) | 0.90 | 0.03  (-0.36, 0.43 ) | 0.02  (2,103.29) | 0.98 | 0.92 | 0.03  (-0.41, 0.46) | 0.93 | -0.02  (-0.41, 0.37) | 0.84 | 0.05  (-0.34, 0.44) |
| Caudal middle frontal (cmᶟ) | 0.37  (2,107.07) | 0.69 | 0.60 | 0.15  (-0.29, 0.59) | 0.39 | | 0.2  (-0.2, 0.59) | 0.78 | -0.06  (-0.46, 0.33) | 0.71  (2,109.76) | 0.91 | 0.87 | -0.05  (-0.49, 0.39) | 0.83 | 0.05  (-0.34, 0.44) | 0.67 | -0.1  (-0.49, 0.3) |
| Cuneus (cmᶟ) | 0.62  (2,136.00) | 0.54 | 0.32 | -0.24  (-0.68, 0.2) | 0.33 | | -0.22  (-0.62, 0.17) | 0.89 | -0.03  (-0.43, 0.37) | 1.41  (2,81.28) | 0.25 | 0.11 | -0.42  (-0.86, 0.03) | 0.17 | -0.34  (-0.73, 0.06) | 0.62 | -0.11  (-0.5, 0.29) |
| Entorhinal (cmᶟ) | 1.32  (2,96.98 | 0.27 | 0.11 | -0.4  (-0.84, 0.04) | 0.42 | | -0.18  (-0.57, 0.22) | 0.32 | -0.23  (-0.62, 0.17) | 1.08  (2,96.01) | 0.34 | 0.15 | -0.4  (-0.84, 0.04) | 0.45 | -0.17  (-0.57, 0.22) | 0.33 | -0.22  (-0.61, 0.18) |
| Fusiform (cmᶟ) | 0.79  (2,108.69) | 0.46 | 0.95 | 0.02  (-0.42, 0.46) | 0.29 | | 0.24  (-0.16, 0.63) | 0.31 | -0.22  (-0.62, 0.18) | 1.22  (2,109.07) | 0.30 | 0.35 | 0.31  (-0.14, 0.75) | 0.12 | 0.36  (-0.03, 0.76) | 0.62 | -0.11  (-0.5, 0.28) |
| Inferior parietal (cmᶟ) | 0.15  (2,136.00) | 0.86 | 0.62 | -0.14  (-0.58, 0.3) | 0.91 | | -0.02  (-0.42, 0.37) | 0.65 | -0.09  (-0.49, 0.3 ) | 0.26  (2,82.48) | 0.77 | 0.50 | -0.2  (-0.63, 0.24) | 0.55 | -0.14  (-0.53, 0.25) | 0.84 | -0.04  (-0.44, 0.35) |
| Inferior temporal (cmᶟ) | 0.25  (2,103.95) | 0.78 | 0.48 | -0.19  (-0.63, 0.25) | 0.72 | | -0.08  (-0.48, 0.31) | 0.67 | -0.1  (-0.5, 0.3 ) | 0.21  (2,107.09) | 0.81 | 0.52 | -0.19  (-0.63, 0.25) | 0.67 | -0.1  (-0.5, 0.29) | 0.73 | -0.08  (-0.47, 0.31) |
| Insthmus cingulate (cmᶟ) | 1.00  (2,107.70) | 0.37 | 0.97 | -0.01  (-0.45, 0.43) | 0.24 | | -0.27  (-0.67, 0.13) | 0.25 | 0.26  (-0.13, 0.66) | 1.50  (2,110.31) | 0.23 | 0.77 | 0.08  (-0.35, 0.52) | 0.25 | -0.28  (-0.68, 0.11) | 0.12 | 0.36  (-0.04, 0.76) |
| Lateral occipital (cmᶟ) | 2,82  (2,101.82) | 0.06 | 0.04 | -0.54  (-0.98, -0.09) | 0.04 | | -0.49  (-0.89, -0.08) | 0.75 | -0.07  (-0.47, 0.32) | 2,49  (2,104.12) | 0.09 | 0.04 | -0.56  (-1.01, -0.11) | 0.05 | -0.48  (-0.88, -0.08) | 0.64 | -0.11  (-0.5, 0.29) |
| Lateral orbitofrontal (cmᶟ) | 0.03  (2,104.75) | 0.97 | 0.93 | -0.02  (-0.46, 0.41) | 0.80 | | -0.06  (-0.46, 0.34) | 0.88 | 0.03  (-0.36, 0.43) | 0.19  (2,107.60) | 0.83 | 0.68 | -0.12  (-0.56, 0.32) | 0.55 | -0.15  (-0.55, 0.24) | 0.89 | 0.03  (-0.36, 0.42) |
| Lingual (cmᶟ) | 1.59  (2,99.95) | 0.21 | 0.18 | -0.36  (-0.8, 0.08) | 0.09 | | -0.4  (-0.81, 0) | 0.81 | 0.05  (-0.34, 0.45) | 1.33  (2,101.90) | 0.27 | 0.26 | -0.33  (-0.77, 0.11) | 0.11 | -0.4  (-0.8, 0) | 0.75 | 0.07  (-0.32, 0.46) |
| Medial orbito frontal (cmᶟ) | 0.04  (2,95.54) | 0.96 | 0.94 | -0.02  (-0.46, 0.42) | 0.85 | | 0.04  (-0.35, 0.44) | 0.78 | -0.06  (-0.46, 0.34) | 0.00  (2,97.83) | 1.00 | 0.99 | 0  (-0.44, 0.44) | 0.98 | 0.01  (-0.39, 0.4) | 0.99 | 0  (-0.4, 0.39) |
| Middle temporal (cmᶟ) | 1.72  (2,96.45) | 0.19 | 0.08 | -0.54  (-0.99, -0.09) | 0.56 | | -0.13  (-0.53, 0.27) | 0.16 | -0.31  (-0.71, 0.09) | 1.56  (2,99.85) | 0.22 | 0.11 | -0.53  (-0.97, -0.08) | 0.64 | -0.11  (-0.5, 0.28) | 0.14 | -0.33  (-0.73, 0.06) |
| Parahippocampal (cmᶟ) | 0.45  (2,107.182) | 0.64 | 0.89 | 0.04  (-0.4, 0.48) | 0.49 | | -0.16  (-0.55, 0.24) | 0.39 | 0.2  (-0.2, 0.6) | 0.78  (2,109.78) | 0.46 | 0.83 | 0.06  (-0.37, 0.5) | 0.41 | -0.19  (-0.59, 0.2) | 0.26 | 0.27  (-0.13, 0.66) |
| Paracental (cmᶟ) | 1.58  (2,85.81) | 0.21 | 0.12 | -0.39  (-0.84 . 0.05) | 0.12 | | -0.35  (-0.75, 0.05) | 0.88 | -0.04  (-0.43, 0.36 ) | 1.18  (2,84.70) | 0.31 | 0.23 | -0.32  (-0.76, 0.12) | 0.14 | -0.35  (-0.74, 0.05) | 0.92 | 0.02  (-0.37, 0.42) |
| Pars opercularis (cmᶟ) | 0.10  (2,84.02) | 0.91 | 0.83 | -0.06  (-0.5 . 0.38) | 0.84 | | 0.04  (-0.35, 0.44) | 0.66 | -0.09  (-0.49, 0.3) | 0.08  (2,83.67) | 0.92 | 0.75 | -0.1  (-0.53, 0.34) | 0.99 | 0  (-0.39, 0.39) | 0.70 | -0.08  (-0.47, 0.31) |
| Pars orbitalis (cmᶟ) | 2,08  (2,110.25) | 0.13 | 0.09 | -0.47  (-0.91 . -0.02) | 0.06 | | -0.43  (-0.83, -0.03) | 0.98 | -0.01  (-0.4, 0.39 ) | 2,48  (2,112,67) | 0.09 | 0.07 | -0.53  (-0.98 , -0.09) | 0.04 | -0.52  (-0.92, -0.12) | 1.00 | 0  (-0.39, 0.39) |
| Pars triangularis (cmᶟ) | 1.14  (2,105.02) | 0.33 | 0.17 | -0.38  (-0.82 . 0.06) | 0.21 | | -0.27  (-0.67, 0.13) | 0.78 | -0.06  (-0.46, 0.33) | 0.88  (2,106.06) | 0.42 | 0.24 | -0.35  (-0.79, 0.09) | 0.24 | -0.27  (-0.66, 0.13) | 0.84 | -0.05  (-0.44, 0.35) |
| Pericalcarine (cmᶟ) | 0.48  (2,95.71) | 0.62 | 0.74 | 0.08  (-0.36 . 0.52) | 0.57 | | -0.15  (-0.54, 0.25) | 0.34 | 0.22  (-0.18, 0.62 ) | 0.45  (2,102,14) | 0.64 | 0.95 | 0.02  (-0.42, 0.46) | 0.49 | -0.19  (-0.58, 0.21) | 0.41 | 0.19  (-0.21, 0.58) |
| Postcentral (cmᶟ) | 3.38  (2,136.00) | 0.04 | 0.04 | -0.51  (-0.95 . -0.06) | 0.02 | | -0.55  (-0.96, -0.15) | 0.95 | 0.01  (-0.38, 0.41) | 3.18  (2,139.00) | 0.04 | 0.06 | -0.49  (-0.93, -0.04) | 0.01 | -0.59  (-1, -0.19) | 0.77 | 0.06  (-0.33, 0.46) |
| Posterior cingulate (cmᶟ) | 1.63  (2,89.03) | 0.20 | 0.72 | -0.09  (-0.53 . 0.34) | 0.10 | | -0.4  (-0.8, 0) | 0.20 | 0.27  (-0.13, 0.67) | 2,09  (2,85.84) | 0.13 | 0.57 | -0.16  (-0.6, 0.28) | 0.06 | -0.47  (-0.87, -0.07) | 0.20 | 0.27  (-0.12, 0.67) |
| Precental (cmᶟ) | 0.22  (2,98.51) | 0.80 | 0.99 | 0  (-0.43 . 0.44) | 0.59 | | -0.13  (-0.52, 0.27) | 0.58 | 0.13  (-0.27, 0.53) | 0.32  (2,102,60) | 0.73 | 0.87 | -0.04  (-0.48, 0.39) | 0.48 | -0.18  (-0.57, 0.21) | 0.58 | 0.13  (-0.27, 0.52) |
| Precuneus (cmᶟ) | 2,10  (2,83.25) | 0.13 | 0.11 | -0.42  (-0.86 . 0.02) | 0.05 | | -0.44  (-0.84, -0.04) | 0.88 | 0.03  (-0.37, 0.43) | 1.48  (2,139.00) | 0.23 | 0.32 | -0.28  (-0.72, 0.16) | 0.09 | -0.4  (-0.8, 0) | 0.55 | 0.12  (-0.27, 0.52) |
| Rostral anterior cingulate (cmᶟ) | 0.47  (2,94.62) | 0.63 | 0.55 | -0.16  (-0.6 . 0.27) | 0.78 | | 0.07  (-0.33, 0.46) | 0.34 | -0.22  (-0.62, 0.18) | 0.35  (2,93.76) | 0.71 | 0.84 | 0.06  (-0.38, 0.5) | 0.45 | 0.19  (-0.2, 0.58) | 0.58 | -0.13  (-0.52, 0.27) |
| Rostral middle frontal (cmᶟ) | 0.10  (2,107.10) | 0.91 | 0.68 | -0.12  (-0.55 . 0.32) | 0.92 | | -0.02  (-0.42, 0.37) | 0.71 | -0.08  (-0.48, 0.31) | 0.15  (2,108.42) | 0.86 | 0.58 | -0.16  (-0.6, 0.28) | 0.74 | -0.08  (-0.48, 0.31) | 0.74 | -0.07  (-0.47, 0.32) |
| Superior frontal (cmᶟ) | 0.63  (2,95.45) | 0.53 | 0.27 | -0.28  (-0.72 . 0.16) | 0.58 | | -0.13  (-0.53, 0.26) | 0.48 | -0.16  (-0.56, 0.24) | 1.09  (2,96.76) | 0.34 | 0.15 | -0.38  (-0.83, 0.06) | 0.27 | -0.29  (-0.68, 0.11) | 0.55 | -0.13  (-0.53, 0.26) |
| Superior parietal (cmᶟ) | 0.04  (2,94.52) | 0.96 | 0.82 | -0.06  (-0.5 . 0.38) | 1.00 | | 0  (-0.4, 0.4) | 0.80 | -0.06  (-0.45, 0.34) | 0.02  (2,98.87) | 0.98 | 0.88 | 0.04  (-0.39, 0.48) | 0.99 | 0  (-0.39, 0.4) | 0.87 | 0.04  (-0.35, 0.43) |
| Superior temporal (cmᶟ) | 1.39  (2,109.17) | 0.25 | 0.10 | -0.45  (-0.9 . -0.01) | 0.25 | | -0.26  (-0.66, 0.13) | 0.50 | -0.15  (-0.55, 0.25) | 1.11  (2,110.29) | 0.33 | 0.15 | -0.43  (-0.87, 0.01) | 0.26 | -0.27  (-0.67, 0.12) | 0.55 | -0.13  (-0.52, 0.26) |
| Supramarginal (cmᶟ) | 2,18  (2,95.69) | 0.12 | 0.04 | -0.52  (-0.97 . -0.08) | 0.31 | | -0.24  (-0.64, 0.16) | 0.19 | -0.28  (-0.67, 0.12) | 2,20  (2,99.53) | 0.12 | 0.04 | -0.57  (-1.01, -0.12) | 0.24 | -0.3  (-0.69, 0.1) | 0.19 | -0.28  (-0.67, 0.11) |
| Frontal pole (cmᶟ) | 0.73  (2,103.53) | 0.49 | 0.59 | -0.13  (-0.57 . 0.31) | 0.56 | | 0.14  (-0.26, 0.53) | 0.24 | -0.29  (-0.69, 0.11) | 0.99  (2,103.15) | 0.38 | 0.59 | -0.14  (-0.58, 0.3) | 0.51 | 0.17  (-0.23, 0.56) | 0.17 | -0.33  (-0.72, 0.07) |
| Temporal pole (cmᶟ) | 1.22  (2,100.84) | 0.30 | 0.13 | -0.4  (-0.84 . 0.05) | 0.29 | | -0.24  (-0.64, 0.16) | 0.52 | -0.15  (-0.54, 0.25) | 1.13  (2,102.96) | 0.33 | 0.14 | -0.41  (-0.86, 0.03) | 0.30 | -0.24  (-0.64, 0.15) | 0.47 | -0.17  (-0.56, 0.23) |
| Transverse temporal (cmᶟ) | 1.47  (2,96.53) | 0.24 | 0.09 | -0.46  (-0.9 . -0.01) | 0.28 | | -0.26  (-0.66, 0.14) | 0.42 | -0.2  (-0.59, 0.2) | 3.36  (2,98.861) | 0.04 | 0.01 | -0.73  (-1.18, -0.28) | 0.05 | -0.48  (-0.88, -0.09) | 0.29 | -0.26  (-0.65, 0.14) |
| Insula (cmᶟ) | 2,83  (2,100.02) | 0.06 | 0.03 | -0.61  (-1.06 . -0.16) | 0.06 | | -0.43  (-0.83, -0.03) | 0.54 | -0.14  (-0.53, 0.26) | 3.34  (2,102.89) | 0.04 | 0.02 | -0.69  (-1.14, -0.24) | 0.02 | -0.55  (-0.95, -0.15) | 0.63 | -0.11  (-0.5, 0.29) |
| Analyses are performed in a mixed model with correction for family membership. Abbreviations: BDo, bipolar disorder offspring; CI, confidence interval; Co, control offspring ; d, Cohen’s d; df, degree of freedom; FDR, false discovery rate; GM, gray matter; SZo, schizophrenia offspring; WM, white matter. The significance level was set at α= 0.05. | | | | | | | | | | | | | | | | | |

| **Table S4. Differences between schizophrenia offspring (SZo), bipolar offspring (BDo) and control offspring after matching the groups on (A) age^a^ and (B) sex^b^.** | | | | | | | | |
| --- | --- | --- | --- | --- | --- | --- | --- | --- |
| **(A) Matched on age** | Main effect | | Pairwise | | | | | |
| **Global brain volumes** |  |  | *SZo < Co* | | *BDo<Co* | | *SZo < BDo* | |
|  | *F (df, df)* | *p* | *p* | *d (CI 95%)* | *p* | *d (CI 95%)* | *p* | *d (CI 95%)* |
|  |  |  | **Effect of GROUP**  **(corrected for age and sex)** | | | | | |
| *Intracranial (cm^3^) ^c^* | 3.53 (2, 99.7) | **0.033** | **0.020** | -0.57  (-1.02, -0.12) | *0.79*  *(BDo > Co)* | -0.07  (-0.48, 0.34) | ***0.024*** | -0.51  (-0.93, -0.09) |
| *Cortical thickness (mm)^c^* | 4.16  (2, 98.5) | **0.018** | **0.005** | -0.73  (-1.18, -0.3) | 0.129 | -0.33  (-0.75, 0.08) | 0.115 | -0.33  (-0.75, -0.08) |
|  |  |  | **Effect of GROUP**  **(corrected for age, sex, and intracranial volume)** | | | | | |
| Lateral ventricles (cmᶟ) | 3.97 (2, 92.5) | **0.022** | 0.126  (SZo > Co) | 0.51  (0.06, 0.96) | **0.006**  (BDo > Co) | 0.59  (0.17, 1.01) | 0.281  (BDo > SZo) | -0.23  (-0.64, 0.18) |
| **(A) Matched on sex** | Main effect | | Pairwise | | | | | |
| **Global brain volumes** |  |  | *SZo < Co* | | *BDo<Co* | | *SZo < BDo* | |
|  | *F (df, df)* | *p* | *p* | *d (CI 95%)* | *p* | *d (CI 95%)* | *p* | *d (CI 95%)* |
| *Intracranial (cm^3^) ^c^* | 1.75  (2, 112.0) | 0.178 | **0.080** | -0.46  (-0.92, -0.01) | 0.67  (BDo > Co) | -0.09  (-0.49, 0.30) | 0.136 | -0.34  (-0.74, 0.07) |
| *Cortical thickness (mm)^c^* | 4.88  (2, 106.7) | **0.009** | **0.003** | -0.77  (-1.24, -0.31) | 0.234 | -0.26  (-0.66, 0.13) | **0.032** | -0.46  (-0.86, -0.05) |
|  |  |  | **Effect of GROUP**  **(corrected for age, sex, and intracranial volume)** | | | | | |
| Lateral ventricles (cmᶟ) | 3.84 (2, 101.5) | **0.025** | 0.113  (SZo > Co) | 0.52  (0.06, 0.97) | **0.007**  (BDo > Co) | 0.58  (0.18, 0.98) | 0.336  (BDo > SZo) | -0.20  (-0.61, 0.20) |
| Analyses are performed in a mixed model with correction for family membership; Abbreviations: BDo, bipolar disorder offspring; CI, confidence interval; Co, control offspring ; d, Cohen’s d; df, degree of freedom; SZo, schizophrenia offspring. ^a^ 13 oldest BDo were removed, groups no longer differed significantly on age (*F*(2,132)=2.75, *p*=0.07, pairwise: BDo v SZo *p*=0.92, BDo v Co *p*=0.07, SZo v Co *p*=0.19); ^b^ 3 female SZo with the smallest ICV were removed, groups no longer differed significantly on sex (Chi-Square=5.58, *p*=0.062); ^c^ Statistics for intracranial volume and mean cortical thickness are not corrected for intracranial volume. The significance level was set at α= 0.05. | | | | | | | | |
