# Supplementary material for: Brain structure, IQ, and psychopathology in young offspring of patients with schizophrenia or bipolar disorder
Source: Eur Psychiatry. 2020 Jan 31;63(1):e5. doi: 10.1192/j.eurpsy.2019.19 (PMC8057400; doi:10.1192/j.eurpsy.2019.19)
Supplement: Supplementary file 1 [file epasup.zip › S0924933819000191sup002.docx]

**Supplementary text**

The automated segmentation procedure of FreeSurfer software package version 5.3.0 (<http://surfer.nmr.mgh.harvard.edu/>) was used for subcortical and cortical volume, cortical thickness, and cortical surface area estimations. This package uses both intensity and continuity information from the entire 3D image volume in segmentation and deformation procedures, to produce a parcellation of the cerebral cortex into 34 ROIs per hemisphere, based on the Desikan-Killiany cortical atlas, gyral curvature, sulcal depth and tessellation of the gray matter (GM)/white matter (WM) boundary (Desikan et al., 2006; Fischl et al., 2004). For cortical and subcortical volumes, an optimal linear transform is computed that maximizes the likelihood of the input image, given an atlas constructed from manually labelled images. A non-linear transform is initialized with the linear one and the image is allowed to deform in order to better match the atlas. Finally, a Bayesian segmentation procedure is performed and the maximum a posterior (MAP) estimate of the labelling is computed (Fischl et al., 2002, 2004). Cortical thickness measures were defined as the closest distance from the GM/WM boundary to the GM/cerebral spinal fluid (CSF) boundary at the location where the greatest shift in intensity defines the transition to the other tissue class (Dale, Fischl, & Sereno, 1999; Fischl & Dale, 2000). Cortical surface area measures were obtained by the generation of a tessellated cortical surface model (Fischl & Dale, 2000). Segmenting intracranial volume from a T1 scan is difficult to do because this image does not highlight the cerebrospinal fluid/skull border, as both are dark on the image. FreeSurfer exploits a relationship between the ICV and the linear transform to MNI305 space (the talairach.xfm; Buckner et al., 2004).

**References**

Buckner, R. L., Head, D., Parker, J., Fotenos, A. F., Marcus, D., Morris, J. C., & Snyder, A. Z. (2004). A unified approach for morphometric and functional data analysis in young, old, and demented adults using automated atlas-based head size normalization: Reliability and validation against manual measurement of total intracranial volume. *NeuroImage*, *23*, 724–738.

Dale, A. M., Fischl, B., & Sereno, M. I. (1999). Cortical surface-based analysis. *Neuroimage*, *207*, 195–207.

Desikan, R. S., Ségonne, F., Fischl, B., Quinn, B. T., Dickerson, B. C., Blacker, D., Buckner, R. L., et al. (2006). An automated labeling system for subdividing the human cerebral cortex on MRI scans into gyral based regions of interest. *NeuroImage*, *31*, 968–980.

Fischl, B., & Dale, A. M. (2000). Measuring the thickness of the human cerebral cortex from magnetic resonance images. *Proceedings of the National Academy of Sciences*, *97*(20), 11050–11055.

Fischl, B., van der Kouwe, A., Destrieux, C., Halgren, E., Ségonne, F., Salat, D. H., Busa, E., et al. (2004). Automatically Parcellating the Human Cerebral Cortex. *Cerebral Cortex*, *14*, 11–22.

Fischl, B., Salat, D. H., Busa, E., Albert, M., Dieterich, M., Haselgrove, C., van Der Kouwe, A., et al. (2002). Whole brain segmentation: automated labeling of neuroanatomical structures in the human brain. *Neuron*, *33*(3), 341–355.
